# Oxidative Stress and Antioxidant Defense Capacity in Patients with Metastatic and Non-Metastatic Cutaneous Melanoma

**DOI:** 10.3390/ijms27188395

**Published:** 2026-09-20

**Authors:** Cinzia Buligan, Gianluca Petris, Giuseppe Stinco, Sabina Cauci

**Affiliations:** 1 Institute of Dermatology, Azienda Sanitaria Universitaria Friuli Centrale (ASUFC), Santa Maria della Misericordia, Friuli Centrale University Healthcare Hospital of Udine, 33100 Udine, Italy; giuseppe.stinco@uniud.it; 2 Department of Medicine, School of Medicine, University of Udine, 33100 Udine, Italy; gianluca.petris@uniud.it (G.P.); sabina.cauci@uniud.it (S.C.); 3 Fondazione Italiana Fegato ETS—Italian Liver Foundation ETS, 34149 Trieste, Italy

**Keywords:** oxidative stress, lipid peroxidation, free radicals, antioxidant defense, cutaneous melanoma, skin cancer, metastasis, stage IV melanoma, gender, smoking, coffee

## Abstract

The role of oxidative stress (OS) and total antioxidant capacity (TAC) in cutaneous melanoma (M) is unclear. We examined 120 healthy controls and 120 M patients, 53 metastatic (MetM) and 67 non-metastatic M (NMetM). Blood OS, and TAC were measured by FORT and FORD rapid tests, respectively. Elevated OS did not differ between M patients and controls, but it was more frequent in MetM patients, with an odds ratio (OR) = 2.51. Low TAC values were more frequent in M patients, MetM, and NMetM than controls; OR = 6.67, OR = 12.4, and OR = 4.53, respectively. High OS combined with low TAC was more frequent in M patients (OR = 3.74) and MetM (OR = 8.48) than controls. Among M patients, elevated OS (n = 46) was more frequent in females (OR = 7.66), Stage IV (OR = 6.68), M1c (OR = 4.87) and ulceration (OR = 2.52), and less frequent in NMetM (OR = 0.28), M0 (OR = 0.15), superficial spreading (OR = 0.46), Breslow thickness < 0.75 mm (OR = 0.33), and epithelioid variant (OR = 0.32). Among M patients, low TAC (n = 87) was more frequent in Stage IV (OR = 20.3), M1c (OR = 1.24), and lower limb (OR = 5.90), and less frequent in NMetM (OR = 0.37). Compared with other M patients, Stage IV patients more frequently had high OS (OR = 6.68), low TAC (OR = 20.3), high OS and low TAC (OR = 7.71), and <5-year mortality (OR = 29.3). This is the largest study investigating systemic OS and TAC in M patients and provides a rationale for further validation of these markers in larger cohorts and different ethnic groups, which might furnish indications for future personalized OS and TAC screening and/or prevention advice.

## 1. Introduction

Melanoma is the most dangerous form of skin cancer and its incidence is rising in most countries, creating a significant clinical problem [1,2,3], although in recent years new therapies have proved to be partially effective with respect to response rate, duration of disease, and overall survival [1,2,3,4]. Cutaneous melanoma, which originates from melanocytes, is highly invasive and metastatic, according to GLOBOCAN 2022 [1]. Melanoma is the 17th most common cancer worldwide, accounting for 1.7% of all new cancer cases, and for 0.6% of all cancer deaths. The incidence of skin cancer varies across geographic regions and countries, with a predominance observed in Oceania, North America and Europe, while in Africa and Asia the incidence rates are very low [1]. Risk factors for the development of cutaneous melanoma include prolonged exposure to ultraviolet (UV) radiation, skin cancer family history, vitamin D related factors, lifestyle, chemical carcinogens, immunity and immunosuppressive agents [1,2,3,4,5,6,7,8,9,10].

An estimated 60–70% of cutaneous malignant melanomas are thought to be caused by UV radiation exposure [1,2,4] from sunlight and/or tanning beds, which cause UV-induced DNA damage, oxidative stress, and inflammation in the skin [4]. UV radiation can induce oxidative DNA damage through direct as well as mediated mechanisms by the formation of 8-hydroxy-2′-deoxyguanosine (8-OHdG) and 8-oxo-7,8-dihydro-2′-deoxyguanosine (8-oxodG), which can cause genetic mutations and, in turn, contribute to skin carcinogenesis [2,4,11].

Oxidative stress (OS) has been implicated in melanoma onset and severity including progression to the metastatic condition [11,12,13]. OS is considered to derive from an imbalance between the amount of free radicals, mainly reactive oxygen (ROS) and reactive nitrogen species (RNS), and counteracting molecules and enzymes called antioxidants able to neutralize the free unpaired electrons [11]. Physiologically, ROS are mainly produced in mitochondria during oxygen-consuming metabolic reactions [11,12,13]. Other endogenous sources of ROS are melanogenesis, cytochrome P450 activities, NADPH oxidase, lipoxygenases, peroxisomes and inflammation [11]. Exogenous sources of ROS include, besides UV radiation, other ionizing radiations, environmental toxins, chemicals, and polluting metals such as Arsenic, Cadmium, and Lead [11,13,14].

In particular, epidermal melanocytes, from which melanoma arises, are constantly exposed to ROS production, including superoxide anion (O_2_^•−^), hydroxyl radical (HO^•^), and hydrogen peroxide (H_2_O_2_), that occurs during melanin biosynthesis, a process that may contribute to melanomagenesis [4,11]. Free radicals are deleterious to the human body because they can retrieve electrons from various molecules (including nucleic acids, lipids, proteins, etc.), provoking formation of oxidized forms, so that severe oxidative stress can even trigger cell death [11]. Recent research demonstrates that ROS can serve as triggers for diverse forms of cell death collectively known as oxidative cell death, such as ferroptosis, apoptosis, necroptosis, and other mechanisms, each displaying distinct genetic, biochemical, and signaling characteristics [11,12,13].

Intracellular ROS detoxification is physiologically mediated by a complex network of non-enzymatic and enzymatic molecules, including vitamins A, C, D, E, reduced glutathione (GSH), ferritin, eumelanin (a brown–black skin pigment that confers photoprotection from solar UV radiation), glutathione peroxidase (GPx), catalase (CAT), superoxide dismutase (SOD), and heme oxygenase 1 (HO-1) [11,12,13,14]. Taken together, they represent the human antioxidant system [11]. UV radiation is known to induce a dose-dependent response by human melanocytes leading to production of H_2_O_2_, but it also leads to decreases in antioxidant CAT activity and a reduction in HO-1 expression [11,15,16,17].

Besides the role of OS in driving malignant transformation of cells into primary tumors, ROS seem to be also involved in cancer progression and metastatic progression [11,17,18,19]. However, paradoxically, in some experimental studies excessive oxidative stress can even have beneficial effects by compromising survival of melanocytes, leading to cell death [13,18,19]. Some evidence in animal models suggests that the use of antioxidants (by limiting oxidative stress) could even promote melanoma metastasis [18]. Thus, in the context of melanoma interventions, both antioxidants and pro-oxidants could have utility, because of their context-dependent ability to promote or suppress tumorigenesis and metastasis [2,4,11,13,17,18,19].

The relationship between melanoma and oxidative stress has been rather extensively studied in melanoma cells in vitro or in animal models [4,11,13,19], but there are few studies that have examined biomarkers of oxidative stress in vivo in melanoma patients [20,21,22,23]. Notably, there is evidence that the data about tumor cells in culture do not correspond to what is observed in humans [19].

In order to better clarify whether OS and the antioxidant defense system (TAC) play a role in melanoma development and progression and to examine a larger population of cutaneous melanoma and healthy subjects than those previously included in human studies, we measured the levels of reactive oxygen species, in the form of blood hydroperoxides (by FORT assay, considered to measure lipid hydroperoxides) and of scavenging molecules (by FORD assay), that orchestrate antioxidant defense. We compared these data between cutaneous melanoma (M) patients and healthy subjects, and between metastatic (MetM) and non-metastatic melanoma (NMetM) patients, and also according to sex and lifestyle.

## 2. Results

Table 1 illustrates characteristics of the 240 study participants and the comparison between the 120 cutaneous melanoma patients (M) and the 120 healthy controls. M patients were enrolled into the study on average 4 years after first melanoma diagnosis. The study subjects were on average 60 years old, with no difference between M cases and controls; most had all four grandparents born in the FVG Region with no difference between groups. M patients had higher BMI values than controls (25.8 ± 3.83 versus 23.5 ± 3.38 kg/m^2^, *p* < 0.001) and were less frequently present smokers (9.2% versus 20.0%, *p* = 0.020), although the number of cigarettes per day did not differ between groups of present smokers. Notably, M patients (40.8%) were almost four-fold more frequently past smokers than healthy controls (15.8%), with OR = 3.67, CI = 1.99–6.76, *p* < 0.001, although number of cigarettes per day did not differ between groups. M patients (50.0%) were more frequently ever smokers than the healthy subjects (35.8%), with OR = 1.79, CI = 1.07–3.00, *p* = 0.027. Interestingly, among ever smokers, M patients (60.0%) more often smoked for 20 or more years than healthy controls (32.6%), with OR = 3.11, CI = 1.37–7.06, *p* = 0.007. The average number of daily cups of Italian espresso coffee did not differ between M patients and healthy controls (*p* = 0.731).

Continuous FORT values and frequency of elevated FORT levels (≥310 FORT units) of hydroperoxides were not different between M patients and healthy controls. In contrast, continuous values of TAC (FORD units) were lower in M patients than controls (1.04 ± 0.21 versus 1.18 ± 0.14 units, *p* < 0.001). Notably, low levels of TAC (<1.10 FORD units) were much more frequent in M patients (72.5%) than in healthy controls, at 28.3% (OR = 6.67, CI = 3.79–11.7, *p* < 0.001).

Finally, the combined condition of elevated OS and low TAC (i.e., ≥310 FORT and <1.10 FORD units) was observed in 35.8% of M patients and in only 11.7% of healthy controls, with OR = 3.74, CI = 1.92–7.29, *p* < 0.001.

Table 2 shows a comparison between metastatic and non-metastatic melanomas, and between each subgroup of M patients and healthy controls. Elevated oxidative stress values (FORT ≥310 units) were much more frequent in MetM (54.7%) than NMetM (25.4%) with OR = 3.55, CI = 1.64–7.69, *p* = 0.001. Low TAC levels (i.e., FORD < 1.10 units) were more frequent in MetM (83.0%) than NMetM (64.2%), with OR = 2.73, CI = 1.14–6.54, *p* = 0.024. The combined condition of high OS and low TAC (FORT ≥ 310 and FORD < 1.10 units) values was more frequent in MetM (52.8%) than NMetM (22.4%), with OR = 3.88, CI = 1.77–8.54, *p* < 0.001.

Furthermore, elevated OS was more frequent in MetM than healthy control, with OR = 2.51, CI = 1.29–4.87, *p* = 0.006. Low TAC was found much more frequently in MetM than in healthy subjects, with OR = 12.4, CI = 5.45–28.1, *p* < 0.001. Notably, the combined condition of high OS and low TAC was much more frequent in MetM than in healthy controls, with OR = 8.48, CI = 3.90–18.4, *p* < 0.001.

NMetM differed from healthy controls only for a higher frequency of low TAC, with OR = 4.53, CI = 2.39–8.58, *p* < 0.001, but NMetM did not differ from healthy controls with respect to high FORT ≥310 units, and only a tendency was observed for NMetM versus healthy subjects regarding the combined condition of elevated OS and low TAC, with OR = 2.18, CI = 0.98–4.86, *p* = 0.056^.

Table 3 shows a comparison of high (FORT ≥310 units) and low (FORT <310 units) OS levels among the 120 M patients. Notably, patients with high OS hydroperoxides (n = 46, 38.3%) were much more likely to be females (OR = 7.66, CI = 3.34–17.6, *p* < 0.001) and to have metastatic melanoma (OR = 3.55, CI = 1.64–7.69, *p* = 0.001), Stage IV (OR = 6.68, CI = 2.23–20.0, *p* < 0.001), staging M1c (OR = 4.87, CI = 1.59–14.9, *p* = 0.003), and ulceration (OR = 2.52, CI = 1.17–5.44, *p* = 0.017), while they were less likely to be males (OR = 0.13, CI = 0.06–0.30, *p* < 0.001), to have NMetM (OR = 0.28, CI = 0.13–0.61, *p* = 0.001), to be in Stage I (OR = 0.31, CI = 0.14–0.71, *p* = 0.005), and to have M0 (i.e., without lymph node or distant metastases) (OR = 0.15, CI = 0.05–0.45, *p* < 0.001), superficial spreading melanoma (OR = 0.46, CI = 0.22–0.98, *p* = 0.043), Breslow thickness <0.75 mm (OR = 0.33, CI = 0.12–0.89, *p* = 0.025), and epithelioid variant (OR = 0.32, CI = 0.13–0.82, *p* = 0.014). Furthermore, some tendencies were observed among M patients: those with FORT ≥ 310 units tended to have ≥4 cups of (espresso) coffee per day, with OR = 2.55, *p* = 0.056^; staging M1a (i.e., metastasis to skin, subcutaneous tissue, or distant lymph nodes), with OR = 1.07, *p* = 0.054^; a nodular melanoma, with OR = 2.12, *p* = 0.054^; a Breslow thickness ≥2.0 and <4.0 mm, with OR = 2.29, *p* = 0.051^; Mitosis ≥1 per mm^2^, with OR = 2.16, *p* = 0.064^; and more than one melanoma, with OR = 2.59, *p* = 0.056^. No significant differences were found regarding: <5-year mortality, age at study entry <50 years, age at first melanoma diagnosis <50 years, regional/geographical background (i.e., four grandparents born in the FVG Region, Northeast Italy), overweight or obesity, education, smoking habits, fair skin phototype 1 and 2, nevi ≥ 50, sunburns ever lifelong, and concurrent skin or non-skin tumor.

Among the 120 M patients, Table 4 shows a comparison of melanoma patients with low (FORD < 1.10 units) (n = 87) and high (FORD ≥ 1.10 units) antioxidant defense capacity (n = 33). A low TAC was a risk factor for metastatic melanoma (OR = 2.73, CI = 1.14–6.54, *p* = 0.024), for Stage IV melanoma (OR = 20.3. CI = 1.19–347, *p* = 0.037) and M1c stage (OR = 1.24, CI = 1.12–1.38, *p* = 0.003) and for having a melanoma located in the lower limbs (OR = 5.90, CI = 1.31–26.6, *p* = 0.011). Patients with low TAC tended to have a Breslow thickness from 2.0 to <4.0 mm (OR = 3.09, *p* = 0.053^) and were slightly less likely to have a small cell variant (OR = 0.94, *p* = 0.075^). It should be noted that low TAC was not associated with sex, as no differences were noted for females and males, in contrast to what was seen in Table 3 for the elevated FORT units.

Patients with melanoma having FORD < 1.10 units had OR = 9.77, CI = 2.77–34.4, *p* < 0.001, with respect to having a condition of FORT ≥ 310 units. In fact, continuous values of FORD were inversely related to FORT units, with r_s_ = −0.426, *p* < 0.001.

Among the 120 M patients, Table 5 shows a comparison of melanoma patients with the combined condition of high OS and low TAC versus all the other remaining M patients. This condition was present in 43 out of 46 patients with high hydroperoxides; thus, the results were similar to those reported in Table 3, but with some differences. Patients with both conditions were much more likely to be females (OR = 5.90, OR = 2.61–13.4, *p* < 0.001), to have MetM (OR = 3.88, OR = 1.77–8.54, *p* = 0.001), to be in Stage IV (OR = 7.71, OR = 2.56–23.2, *p* < 0.001), and to have visceral, non-pulmonary metastases (M1c) (OR = 5.57, CI = 1.81–17.2, *p* = 0.001), nodular melanoma (OR = 2.17, CI = 1.00–4.72, *p* = 0.048), a Breslow thickness between 2 and <4 mm (OR = 2.67, CI = 1.14–6.22, *p* = 0.021), ulceration (OR = 2.24, CI = 1.04–4.85, *p* = 0.039), skin metastases (M1a) (OR = 13.4, CI = 0.68–2.66, *p* = 0.089^) and more than one melanoma (OR = 2.29, CI = 0.85–6.17, *p* = 0.096^); while they were less likely to be males (OR = 0.17, CI = 0.07–0.38, *p* < 0.001), in Stage I (OR = 0.31, CI = 0.14–0.72, *p* = 0.005), and to have M0 (OR = 0.13, CI = 0.04–0.39, *p* < 0.001), a Breslow thickness <0.75 mm (OR = 0.29, CI = 0.10–0.83, *p* = 0.016), and an epithelioid variant (OR = 0.29, CI = 0.11–0.78, *p* = 0.011). M patients with the combined condition (high OS and low TAC) had a tendency to be less likely to have superficial spreading (OR = 0.50, CI = 0.23–1.05, *p* = 0.064^) and more likely to have Mitosis ≥1 per mm^2^ (OR = 2.20, CI = 0.95–5.13, *p* = 0.064^).

Finally, among the 120 M patients, we compared patients with a Stage IV melanoma (n = 20), which poses a high risk of severe prognosis [1,2,3,4], versus all the other M patients (i.e., Stage I, II, and III) (n = 100). Continuous values of OS were much higher in Stage IV patients than in other M patients (mean ± SD, 353.4 ± 64.7 versus 287.4 ± 62.6 FORT units, *p* < 0.001), whereas TAC values were lower (mean ± SD, 0.96 ± 0.11 versus 1.06 ± 0.22 FORD units, *p* = 0.047). Table 6 illustrates the differences in terms of categorical variables between M patients having a Stage IV melanoma and all the other M patients. As many as 75% of Stage IV patients had high OS versus 31% of other M patients (OR = 6.68, CI = 2.23–20.0, *p* < 0.001), all (100%) had low TAC versus 67% of other M patients (OR = 20.3, CI = 1.19–347, *p* = 0.037), and 75% had the combined condition (high OS and low TAC) versus 28% of other M patients (OR = 7.71, CI = 2.56–23.2, *p* < 0.001). As expected [1,2,3,4], Stage IV patients had a high mortality rate within 5 years from present study entry (80%) compared to other M patients (12%), with OR = 29.3, CI = 8.40–102, *p* < 0.001.

No differences were noted with respect to anthropometry, sex, and/or lifestyle characteristics, with the exception of education, which was less likely to be of high school/graduation level in Stage IV patients, with OR = 0.30, CI = 0.11–0.84, *p* = 0.022. Regarding melanoma anatomical site, no differences were noted, with the exception of head and neck, which had a tendency to be more frequent in Stage IV than in other melanomas, with OR = 3.32, CI = 0.87–12.7, *p* = 0.079^. Almost half (45%) of Stage IV patients showed a Breslow thickness from ≥2.0 to <4.0 mm, versus 21% of the other M patients, with OR = 3.08, CI = 1.13–8.40, *p* = 0.028. As many as 65% of Stage IV patients had ulceration versus 21% of other M patients, with OR = 6.99, CI = 2.48–19.7, *p* < 0.001; and 16.7% had microsatellitosis versus 2.0% of other M patients, with OR = 8.65, CI = 1.34–55.6, *p* = 0.023. The epithelioid variant was less frequent in Stage IV patients (5.3%) than in others (33%), with OR = 0.11, CI = 0.01–0.83, *p* = 0.033. Non-brisk TILs tended to be less frequent in Stage IV patients than other M patients, with OR = 0.28, CI = 0.08–1.00, *p* = 0.051^.

## 3. Discussion

We present new information on systemic OS and TAC in cutaneous melanoma patients, demonstrating that a combined phenotype characterized by increased oxidative stress and reduced antioxidant capacity is strongly associated with metastatic disease and advanced tumor stage.

Recently, scientific interest in the role of oxidative stress in melanoma has increased, both for maintaining health and for disease progression implications [2,11,19]. However, discrepancies still exist across cancer studies, particularly regarding proposed use of pro- or anti-oxidative stress strategies, which appear to be highly context-dependent [19,24,25].

### 3.1. Oxidative Stress Evaluated by Lipid Peroxidation (FORT Units)

Our cross-sectional study explored continuous and categorized blood oxidative stress levels according to metastatic and non-metastatic melanoma in a sample of 120 ethnically homogeneous Caucasian patients from a geographical Italian Region (Friuli-Venezia Giulia) with a high incidence rate of cutaneous melanoma [3,26], and 120 healthy controls. We found that OS levels vary considerably among melanoma patients according to metastatic grade; by evaluating stratified high levels of lipid peroxidation (≥310 FORT units), we observed more elevated frequencies for MetM compared to NMetM (OR = 3.55, *p* = 0.001). Notably, Stage IV melanoma had higher OS than other M patients, both considering continuous values of FORT units (*p* < 0.001) and stratified levels (≥310 FORT units) (75% versus 31%, OR = 6.68, *p* < 0.001). In contrast, we did not observe increased high levels of OS in NMetM compared to healthy subjects.

In our M patients elevated OS was not associated with elevated BMI; this suggests that oxidative stress increase may involve mechanisms that are not primarily driven by BMI-related factors [27].

In melanoma, high levels of oxidative stress have been attributed to intrinsic and extrinsic factors, including melanocyte ROS generation related to melanin synthesis, ROS produced within the tumor microenvironment (including by immune cells), and UV radiation exposure [11,12,13,28]. For these reasons, melanocytes and melanoma cells feature unique redox regulation and are characterized by their particular ROS-generating roles [28]. In fact, several studies demonstrated that oxidative stress is increased in melanoma cells in vitro [11,13], although findings observed in cultured cancer cells often do not reflect what happens in patients [11], where redox balance may be modulated by multiple biological and environmental factors, including lifestyle (particularly smoking habits and exposure to pollutants, open air light exposure, alimentary habits), tumor family history, previous exposure to sunburns, concurrent tumor and non-tumor diseases, drug use, DNA mutations (mainly BRAF, NRAS and NF1 genes), geographical area, and genetic background [1,2,3,4,6,7,8,9,11,19,20,21,22,23,25,29,30,31,32].

Regarding the few studies on humans related to the topic of melanoma and oxidative stress, in 2015, a Brazilian study [21] examined 43 patients (15 males and 28 females) with cutaneous melanoma (divided into two groups according to Breslow thickness <2 mm or >2 mm) after surgical tumor removal and 50 (11 males and 39 females) healthy volunteers. Cutaneous melanoma patients had higher MDA (which is a lipid peroxidation end-product) and total thiol levels, lower GSH, and unchanged total radical-trapping antioxidant parameter (TRAP) and uric acid as compared to controls. That study [21] suggested a correlation between Breslow thickness and a systemic pro-oxidant status, similar to our study, where the combined condition of high FORT and reduced TAC was associated with elevated Breslow thickness (between 2 and 4 mm), with OR = 2.67, *p* = 0.021. Furthermore, in our melanoma population, other histological features of increased metastatic risk, such as ulceration (OR = 2.52, *p* = 0.017) and Mitosis ≥1 per mm^2^ (OR = 2.16, *p* = 0.064^, a tendency), were associated with high OS. In a subsequent study, the same Brazilian authors [22], by comparing 30 melanoma patients and 30 controls, found that patients with a metastatic melanoma had less circulating TGF-β1 and increased lipid peroxidation, advanced oxidation protein products (AOPP), TRAP, and thiol levels. MDA was increased in both melanoma groups (MetM and NMetM), while catalase, GSH, and IL-1β were decreased in NMetM. Those findings reinforce the correlation between OS and metastatic melanoma, as in our study group.

In a study performed in Belgrade, Serbia, by Bisevac and colleagues [23], 72 melanoma patients (of which five were in Stage IV) and 30 healthy controls having a mean age of 55 years were examined for serum OS and antioxidant enzyme activity values. Concentrations of superoxide anion were higher in M patients than in controls; also, MDA was higher in M patients than in controls, with the highest values found in the Stage IV group [23]. Those results aligned with the elevated lipid hydroperoxides we found in MetM, particularly in our Stage IV patients.

Oxidative stress associated with melanoma stages in humans is still poorly studied and understood. Overall, among our findings, it was remarkable that elevated systemic OS was associated with metastatic stage in vivo. We cannot infer, however, whether metastatic cells over-produce ROS favoring their proliferation or whether a pro-oxidant environment favors the settlement of the metastatic process. An animal study [18] of mice transplanted with melanomas derived from four patients reported that cytoplasmic ROS levels were significantly high in circulating melanoma cells, suggesting that metastatic dissemination may be accompanied by increased ROS burden [18]. Another study showed that ROS favor oncogene actions that fuel aberrant tumor cell proliferation [33].

These findings raise the possibility that the increased blood ROS observed in metastatic melanoma patients could be associated with processes linked to metastatic disease; however, our data do not identify their cellular source. It also remains unclear whether systemic redox alterations contribute to metastatic dissemination or instead reflect the biological consequences of advanced disease. Many other mechanisms contribute to metastatic dissemination: melanoma cells that survive the circulatory system and reach different organs/tissues interact with the vascular endothelium to begin secondary colonization. The interaction of melanoma and endothelial cells in capillary beds, a critical step in the initiation of metastasis, involves mechanical contact and transient adhesion. This interaction initiates a cascade of activation pathways involving cytokines, growth factors, bioactive lipids, ROS produced by melanoma cells and the endothelium. Immune cells are also present in the metastatic microenvironment [12].

FORT and FORD measurements alone cannot determine whether systemic oxidative stress is a cause or a consequence of metastatic disease; rather, our findings support an association between systemic redox imbalance and metastatic melanoma.

### 3.2. Total Antioxidant Capacity (TAC) (FORD Units)

Several detoxification mechanisms and scavenging molecules orchestrate the body’s antioxidant defense [11]. We found an inverse relationship of lipid peroxidation with antioxidant defense, consistent with previous observations [11,13,17,19,23].

An important finding of our study was that melanoma patients showed markedly decreased antioxidant defense (<1.10 FORD units) as compared to healthy subjects (72.5% versus 28.3%, OR = 6.67, *p* < 0.001). This effect was more pronounced for MetM compared to healthy controls (OR = 12.4, *p* < 0.001) than for NMetM (OR = 4.53, *p* < 0.001) compared to healthy controls. Importantly, all Stage IV patients had low TAC. Among melanoma patients with high OS, only three out of 46 did not have low TAC; in other words, only 6.5% of them retain a non-reduced antioxidant defense. The present study cannot define FORT or FORD thresholds predictive of metastatic progression, which may also vary according to patient characteristics, including sex [34,35,36].

A number of studies evidenced that in melanoma some factors contributing to TAC are dampened. Our current findings are partially in line with a previous Serbian study [23] examining three serum antioxidant enzymes—tSOD, manganese SOD (MnSOD), and CAT activity—besides oxidative stress in blood of patients by dosage of superoxide anion O_2_^•−^ and MDA. In that study, activity of tSOD and MnSOD was not statistically different in a comparison of 72 melanomas with 30 controls, although the subgroup of Stage III patients had higher tSOD and the subgroup of Stage IV patients had higher MnSOD than controls [23]. At variance, serum CAT activity was higher in melanomas than controls, but not in Stage IV patients as compared to controls [23]. Those authors found an inverse relationship of CAT, tSOD, and MnSOD with superoxide anion; thus, their results fit with our observation of a strong inverse relationship between OS and TAC in M patients. It is interesting to note that for Stage IV patients the Bisevac et al. [23] study observed elevated superoxide anion but unchanged tSOD and CAT; those results appear to concur with our finding of elevated OS and low TAC in Stage IV patients.

Our findings partially concur with a Bulgarian study performed on 21 melanoma patients showing higher plasma MDA and lower erythrocyte SOD, although in parallel with increased plasma CAT activity in comparison to control healthy volunteers [20].

Another component of antioxidant defenses is reduced glutathione (GSH), a key factor necessary for transformation of highly pro-oxidant H_2_O_2_ in harmless H_2_O. Piskounova and colleagues [18] found that the ratio of GSH to oxidized glutathione (GSSG) was significantly lower in metastatic nodules or circulating melanoma cells as compared to subcutaneous tumors in melanoma-transplanted mice. This finding is also in line with our observation of low TAC in melanoma patients.

It is important to underline, however, that our data do not permit an inferring of causality; i.e., the present study cannot assess whether melanoma directly increased ROS production, which provoked lipid peroxidation and consequently consumed antioxidant defenses, and/or whether melanoma directly reduced antioxidant defenses, which became insufficient to further neutralize free radicals, in turn causing hyper lipid peroxidation, or whether both mechanisms contribute.

In fact, the biology of oxidative stress in melanoma remains to be elucidated. Overall, human studies generally show increased lipid peroxidation markers in melanoma, while antioxidant measures can be conflicting across studies, possibly depending on the specific markers assessed (enzymatic versus non-enzymatic antioxidants) and disease context. The FORD test provides a global measure of total antioxidant capacity, and it remains to be determined which specific molecules and/or enzymes contribute most to the low TAC observed in our melanoma patients. Evidence also suggests that redox balance may vary with tumor progression [19].

A possible general explanation is provided by the review of Cannavò et al. [11] on the role of oxidative stress in the biology of melanoma. This review examined 29 studies, concluding that increased ROS in tumor cells has a dual effect: on the one hand, they promote tumor survival, proliferation and metastasis, and on the other, at excessive levels, they induce apoptosis and tumor senescence through DNA damage. In this view, melanoma may display adaptive mechanisms that allow tumor cells to tolerate high ROS levels while maintaining redox homeostasis within a range compatible with survival and dissemination [11].

Several studies have examined strategies aimed at reducing oxidative load through antioxidant use. However, the effects of antioxidant interventions in melanoma are context-dependent, and their preventive or therapeutic utility remains uncertain [17,19,24,25].

A greater understanding of redox mechanisms in melanoma and their relationship to molecular and behavioral factors may help define the potential clinical relevance of systemic oxidative measurements. Prospective studies are required to determine whether integrating redox measures with genetic biomarkers and behavioral risk factors can improve prevention or treatment monitoring [32].

Notably, in our study elevated OS and markedly reduced TAC was observed, on average, 4 years after melanoma excision, suggesting persistent systemic alterations associated with melanoma history. Whether these changes reflect long-lasting biological reprogramming, including possible epigenetic contributions, remains to be determined [11,17,32].

### 3.3. Sex Effects

In our study among melanoma patients with high OS, 69.6% were females versus 23.0% females among M patients without high OS (OR = 7.66, *p* < 0.001). This finding was unexpected. At variance, no sex differences were observed for low TAC, nor for Stage IV among melanoma patients. Our study was not designed to assess sex differences; therefore, we cannot provide a definitive explanation.

Sex differences in melanoma incidence, anatomic distribution, and outcomes have been reported, and recent studies confirm consistent patterns across populations [34,35,36]. In women, melanoma incidence has historically been reported to be highest on lower limbs in most age groups in all populations [34,35,36], while in men the melanoma incidence, which increases with age, was always highest on the trunk (from about age 25 years) and head/neck (from about age 40 years). UV radiation exposure changes have been implicated in sex differences, as well as innate differences between the sexes in site-specific susceptibility [34]. In general, in early life (up to approximately 45 years of age) women experience higher rates of melanoma than men, while men develop higher rates of melanoma later in life (from over 65 years of age) [34]. Hormonal factors, including oral contraceptive use, hormone replacement therapy and fertility treatments, have been studied to assess sex disparities in melanoma [35,36,37,38,39,40]. Sex-specific genetic roles in nevi distribution [41], and genetic and epigenetic mutations for melanoma survival [42], have been studied. Of interest, it was recently shown that pregnant women diagnosed with melanoma have a higher overall survival rate than non-pregnant women [43,44], and that in general women have a survival benefit in comparison to men [39,40,45], suggesting possible biological/hormonal sex differences in melanoma tumor–host interactions. So far, however, no clear definitive comprehensive explanations for sex differences in melanoma exist [34,45]. It should be noted that in our present study women taking any hormonal drug were not enrolled, because recent evidence showed that external hormones, including oral contraception, can increase oxidative stress in females [46,47,48]; thus, we can exclude our present results about sex differences in OS having been affected by current use of external hormones. However, recent reviews [39,40,] found that melanoma risk is associated with past use and duration (over 5 years) of use of external hormone treatments; thus, these aspects should be examined in future studies focused on OS and TAC in female melanoma patients. Overall, our findings add to the notion that sex differences may be relevant when interpreting systemic oxidative stress patterns in melanoma and support further studies of sex-specific redox phenotypes, particularly to improve OS screening and prevention advice for women [35,36,40,47,48].

### 3.4. Strengths and Weaknesses

A strength of our study is the geographically restricted area where patients and healthy controls were enrolled; in fact, all of them were Caucasian patients living in Friuli-Venezia Giulia, an Italian Region in Northeast Italy, most had all four grandparents born in the same FVG Region, and indeed the majority were living in a neighborhood of the city of Udine. This supports a relatively similar genetic background and partially shared environmental exposures [8,9,49]. On the other hand, our results should be confirmed in other kinds of populations.

Regarding oxidative stress evaluation, we used a rapid test (FORT assay) that measures hydroperoxides in blood. It is widely known that these molecules are only one type (although one of the most reliable) [11] of the end products resulting from free radicals’ attacks. Thus, other biomarkers of OS should be used to confirm our findings.

Regarding antioxidant defense, the FORD assay provides a rapid global measure of the blood’s total capacity to neutralize free radicals. The specific molecules and/or enzymes underlying the low TAC observed in melanoma patients cannot be identified from the present data.

A limitation of our study was that independent biomarkers of oxidative damage and antioxidant defense were not assessed. For example, assessment of lipid peroxidation products such as MDA or 4-HNE, the GSH/GSSG redox balance, and antioxidant enzymes including SOD, catalase and glutathione peroxidases, together with analysis of pathways involving NRF2–KEAP1 and the glutathione/GPX4 antioxidant system, could provide orthogonal biochemical validation of the FORT/FORD observations.

A further limitation was that the study was not designed to assess the effects of melanoma therapies on oxidative status. Therefore, potential treatment-related effects on FORT and FORD measurements cannot be excluded.

For future studies it will be interesting to verify whether elevated OS and low TAC are correlated to the inflammatory response [7,8,9], and to levels of factors such as vitamin D. In this line, recent studies found that in healthy women FORT units strongly correlate with C-reactive protein concentrations [47,48]. Moreover, our previous studies found influences of vitamin D-related genes, specifically vitamin D receptor (VDR) polymorphisms [5,6] and innate immunity genes, specifically interleukin 1 receptor antagonist (*IL1RN*) and interleukin-1β gene (*IL1B*) polymorphisms in melanoma [7,8,9].

Finally, in our study some confidence intervals were wide and certain metastatic subgroups were limited; larger studies will be needed to refine risk estimates and evaluate additional metastatic categories (i.e., M1b and M1d).

## 4. Methods and Materials

### 4.1. Participants and Study Design

Study participants were consecutively enrolled for a case–control study approved by the Udine Institutional Ethical Committee, in accordance with the Declaration of Helsinki. All subjects provided signed written informed consent before entering the study. Enrollment and clinical visits of all study participants were performed at the Udine University Institute of Dermatology between 2019 and 2024. Diagnostic procedures were carried out according to routine protocols [7,8,9].

The study enrolled 120 (age range of 31–87 years) unrelated patients (hospitalized or outpatients) of both sexes with documented cutaneous melanoma diagnosis who underwent surgery for primary melanoma removal. Controls were 120 (age range of 30–90 years) asymptomatic healthy subjects of both sexes, who were matched for age, geographical area, and similar socioeconomic status with melanoma cases. Inclusion criteria for both melanoma cases and healthy controls were as follows: Caucasian residents in the Friuli-Venezia Giulia (FVG) Region (located in Northeast Italy, at the border with Austria and Slovenia) with at least two Italian grandparents born in the FVG Region (or Austro-Hungarian territory before World War I), as described [6,7,8,9]. Due to the demonstrated association of contraceptive pill use with elevated oxidative stress [46,47,48], women currently using exogenous hormonal treatments were excluded both from melanoma cases and healthy controls. Further, exclusion criteria for healthy controls included the following: any kind of lifelong malignant or benign tumor, first-degree relatives with a history of melanoma, and acute diseases (such as current/recent infection) or chronic diseases, such as severe autoimmune diseases, including type 1 diabetes [7,8,9,50].

Melanoma was diagnosed using immunohistological findings obtained after surgical excision of nevi with clinical and dermoscopic characteristics suggesting the presence of malignancy [7,8,9]. Classification of melanoma stages was performed by clinical/histological/radiological findings following the AJCC 8th edition, as described [8,9,51]. In the TNM classification, M0 defines the condition of no distant metastases (including Stages I, II, and III), M1a of distant skin, subcutaneous, or nodal metastases, M1b of lung metastases, M1c of all other visceral metastases, and M1d of distant metastasis to the central nervous system (CNS) with or without M1a, M1b, or M1c sites of disease [51]. Inclusion criteria for case patients comprised only cutaneous, not mucosal, melanomas. For patients with multiple melanomas, the major melanoma characteristics were accounted for in study analyses according to the histological assessment of major primary tumor (T) grading. All non-metastatic melanoma patients have been followed for at least 5 years to assess absence of metastasis development.

Each participant answered a questionnaire, which was used to collect data on demographic characteristics, medical and family history of melanoma, smoking habits, and lifestyle data, as described [8,9]. Phototype was assessed by Fitzpatrick criteria [52]. BMI was determined by weight (kg) divided by squared height (m^2^); BMI ≥ 25 kg/m^2^ was considered as overweight condition and BMI ≥ 30 kg/m^2^ was considered an indicator of obesity [6,8,9].

### 4.2. Oxidative Stress and TAC Evaluation

Capillary blood samples (20 µL) were obtained from seated and 2 h fasting subjects in the morning (between 8 and 10 am) [46,50]. Personnel executing the collection and measurement of samples were blinded to clinical, demographic, and habit data. Reactive oxygen species, in the form of lipid hydroperoxides, were determined as previously described [46,47] using the Free Oxygen Radical Test (FORT) rapid assay (Callegari, Parma, Italy), a 6 min colorimetric assay based on the ability of transition metals to catalyze the breakdown of hydroperoxides (ROOH) (mainly lipid peroxides) into radicals, according to the Fenton reaction. Results were expressed as FORT units, whereby 1 FORT unit corresponded to 0.26 mg/L H_2_O_2_. The intra- and inter-assay variations were both <5.0%. Detection limits of the assay were ≤160 and ≥600 FORT units. No samples had values over the upper detection limit; only 1 sample (a healthy control) had FORT values below the lower detection limit and was set at 160 FORT units for statistical calculations. Stratification of lipid peroxidation was performed by the threshold of 310 FORT units as a high level, as indicated by the manufacturer and as previously adopted [46,47,50].

A further blood capillary sample (50 µL) to determine the Free Oxygen Radical Defense (FORD) test (Callegari, Parma, Italy), evaluating total antioxidant capacity (TAC), was drawn [48]. The FORD assay uses a preformed stable colored radical cation photometrically detectable at 505 nm; antioxidant compounds in the sample reduce the radical cation, quenching the color and producing a discoloration of the solution, which is proportional to the concentration of antioxidants in the sample. The absorbance values obtained for the sample are compared with a standard curved obtained using 6-hydroxy-2,5,7,8-tetramethylchroman-2-carboxylic acid (Trolox), a soluble derivative of vitamin E commonly employed as an antioxidant. FORD units were mmol/L Trolox equivalents [46,48]. Intra- and inter-assay variations were both <5.0%. Detection limits of the assay were ≤0.25 and ≥3.00 FORD units. No samples had values outside the linear range of FORD detection. Normal interval of values, according to the manufacturer, Callegari, was 1.10–1.50 FORD units. Thus, stratification of oxygen radical defense was performed according to the threshold of <1.10 FORD units as a low level of TAC [46]. The FORD test is an indirect measure of total serum antioxidant capacity (TAC), with ascorbic acid, total thiols (including glutathione), proteins like albumin and ceruloplasmin, bilirubin, polyphenols like flavonoids and tannins (but not uric acid) accounting for the majority of antioxidant activity [47,53].

The repeatability (i.e., analytical imprecision coefficient of variation, CVA) of these rapid tests, 3.9% for FORT and 3.7% for FORD, is comparable with that reported for laboratory measures of OS [48,53].

### 4.3. Statistical Analysis

Continuous data were presented as mean and standard deviation (±SD). The unpaired t-test for independent samples was used for comparison of continuous variables. Correlation between continuous values of FORT and FORD units was calculated using Spearman’s rho coefficient (r_s_). The differences in proportions between categorized groups was assessed by Pearson’s χ^2^-test or Fisher’s exact test, as appropriate. Odds ratios (ORs) and 95% confidence intervals (CIs) were evaluated for categorical variables. With an alpha level of 0.05, the sample size was adequate to detect differences in systemic oxidative stress between melanoma cases and healthy controls, and between subgroups of M patients. All tests were 2-sided. *p* values ≤ 0.050 were considered statistically significant; *p* values ≤ 0.100 were considered indicative of a tendency and denoted by superscript ^. Statistical analysis was performed using the Statistical Package for Social Sciences (SPSS for Windows, SPSS Inc., Chicago, IL, USA), version 28.

## 5. Conclusions

The role of oxidative stress in melanoma is still enigmatic. One main finding of our study was the elevated OS levels in metastatic M patients, particularly those in Stage IV, among 120 Caucasian M patients. As Stage IV patients have a poor survival rate, our findings appear to be of relevance.

The second main finding was the observation of low systemic antioxidant defense capacity in most M patients; in this respect, they largely differed from healthy controls. Low TAC was even more frequent in MetM than in NMetM. Notably, all Stage IV M patients had low TAC; in other words, they are likely to be unable to further counteract ROS/RNS attacks at systemic levels. We cannot, however, infer what is happening inside the tumor microenvironment, where elevated ROS levels may be potent triggers of cell death.

To our knowledge, this is the first study in patients to show a markedly higher frequency of low TAC levels in melanoma patients than in healthy subjects, even several years (on average 4 years) after initial diagnosis and primary tumor excision, in both metastatic and non-metastatic patients. These persistent differences may reflect long-lasting biological changes, but their underlying mechanisms cannot be determined from the present data. The association of oxidative stress biomarkers with smoking duration and the potential contribution of genetic background warrant further investigation. Reduced basal antioxidant defenses could increase susceptibility to pro-oxidative conditions and may have implications for skin cancer development, including melanoma progression.

Further research is warranted to clarify biochemical pathways of oxidative stress and antioxidant defense in melanoma also according to sex.

In future studies, it will be interesting to verify if success of current therapies for advanced melanoma is modulated by oxidative stress and/or TAC blood levels.

The rapid FORT and FORD tests are practical to perform, but their potential clinical utility at diagnosis or during follow-up requires prospective validation, including assessment of whether changes in these measures are associated with treatment response or clinical outcomes.

The relationship between environmental/lifestyle pro-oxidant exposures, systemic redox status, and melanoma outcomes should be assessed in prospective studies [54,55]. The potential interaction between exogenous hormonal treatments, systemic oxidative status, and melanoma outcomes also warrants dedicated investigation in female patients. Sex-specific studies including also detailed gynecologic history are warranted to take into account sex differences in factors potentially modulating melanoma and oxidative stress. Future studies should also assess whether individual specific genetic variations might affect oxidative status [56].

An improved understanding of systemic redox alterations in melanoma may guide future mechanistic and translational studies of metastatic disease.

## Figures and Tables

**Table 1:** Characteristics, OS hydroperoxides (FORT units) and total antioxidant defense capacity (FORD units) values in all 240 study subjects, and comparison between 120 cutaneous melanoma patients and 120 healthy controls.

Characteristic	All Subjects (n = 240)	Melanoma Patients (n = 120)	Healthy Controls (n = 120)	OR (CI), Melanomas vs. Healthy	*p*-Value Melanomas vs. Healthy
Age, years, mean ± SD	60.0 ± 13.0	61.3 ± 11.9	58.6 ± 13.9	**-**	0.108
All grandparents born in the FVG Region, n (%)		88 (73.3)	77 (64.2)	1.54 (0.89–2.66)	0.127
BMI, kg/m^2^ mean ± SD	24.7 ± 3.75	25.8 ± 3.83	23.5 ± 3.28	**-**	<0.001
Time from first diagnosis to study enrollment, years, mean ± SD	-	4.0 ± 3.7	-		
Present smoker, n (%)	35 (14.6)	11 (9.2)	24 (20.0)	0.40 (0.19–0.87)	0.020
Number cigarettes/day in present smokers, mean ± SD	9.67 ± 7.03	12.5 ± 6.89	8.35 ± 6.83	-	0.102
Past smokers, n (%)	68 (28.3)	49 (40.8)	19 (15.8)	3.67 (1.99–6.76)	<0.001
Number cigarettes/day in past smokers, mean ± SD	15.7 ± 11.7	15.2 ± 10.2	17.2 ± 15.0	-	0.532
Ever smoker, n (%)	103 (42.9)	60 (50.0)	43 (35.8)	1.79 (1.07–3.00)	0.027
≥20 years smoking in ever smokers, n (%)	50 (48.5)	36 (60.0)	14 (32.6)	3.11 (1.37–7.06)	0.007
Number of coffee (espresso) cups/day, mean ± SD	2.31 ± 1.54	2.35 ± 1.48	2.28 ± 1.61	-	0.731
Hydroperoxides (OS), FORT units, mean ± SD	293.5 ± 63.3	298.4 ± 67.4	288.5 ± 58.7	-	0.228
High hydroperoxides (OS), FORT ≥ 310 units, n (%)	85 (35.4)	46 (38.3)	39 (32.5)	1.29 (0.76–2.19)	0.345
Total antioxidant capacity, FORD units, mean ± SD	1.11 ± 0.19	1.04 ± 0.21	1.18 ± 0.14	-	<0.001
Low total antioxidant capacity, FORD < 1.10 units, n (%)	121 (50.4)	87 (72.5)	34 (28.3)	6.67 (3.79–11.7)	<0.001
FORT ≥ 310 units and FORD < 1.10 units, n (%)	57 (23.7)	43 (35.8)	14 (11.7)	3.74 (1.92–7.29)	<0.001

**Table 2:** Comparisons of categorical high levels of hydroperoxides (FORT ≥ 310 units) and low total antioxidant defense capacity (FORD < 1.10 units) values in metastatic (MetM) and non-metastatic (NMetM) melanoma patients, and healthy subjects.

Characteristic	MetM Patients (n = 53)	NMetM Patients (n = 67)	MetM vs. NMetM OR (eliminate 95% CI), *p* Value	MetM vs. Healthy OR (CI), *p* Value	NMetM vs. Healthy OR (CI), *p* Value
High hydroperoxides (OS), FORT ≥ 310 units, n (%)	29 (54.7)	17 (25.4)	3.55 (1.64–7.69), 0.001	2.51 (1.29–4.87), 0.006	0.71 (0.36–1.38), 0.309
Low antioxidant defense capacity, FORD < 1.10 units, n (%)	44 (83.0)	43 (64.2)	2.73 (1.14–6.54), 0.024	12.4 (5.45–28.1), <0.001	4.53 (2.39–8.58), <0.001
FORT ≥ 310 units and FORD < 1.10 units, n (%)	28 (52.8)	15 (22.4)	3.88 (1.77–8.54), <0.001	8.48 (3.90–18.42), <0.001	2.18 (0.98–4.86), 0.056 ^

Tendencies are evidenced with superscript ^.

**Table 3:** Clinical characteristics of 120 melanoma patients, and comparison between the two subgroups with high hydroperoxides (OS) (FORT ≥ 310 units (n = 46)) and low hydroperoxides (FORT < 310 units (n = 74)).

Characteristic	All Melanoma Patients (n = 120)	FORT ≥ 310 Units (n = 46)	FORT < 310 Units (n = 74)	FORT ≥ 310 vs. <310 Units OR (CI)	FORT ≥ 310 vs. <310 Units *p* Value
Dead within 5 years from study entry, n (%)	28 (23.3)	13 (28.3)	15 (20.3)	1.55 (0.66–3.65)	0.316
Age at study entry <50 years, n (%)	21 (17.5)	8 (17.4)	13 (17.6)	0.99 (0.37–2.60)	0.980
Age at first melanoma diagnosis <50 years, n (%)	37 (30.8)	15 (32.6)	22 (29.7)	1.14 (0.52–2.53)	0.740
Females, n (%)	49 (40.8)	32 (69.6)	17 (23.0)	7.66 (3.34–17.6)	<0.001
Males, n (%)	71 (59.2)	14 (30.4)	57 (77.0)	0.13 (0.06–0.30)	<0.001
All grandparents born in FVG, n (%)	88 (73.3)	34 (73.9)	54 (73.0)	1.05 (0.46–2.42)	0.910
BMI ≥ 25, kg/m^2^, n (%)	63 (52.5)	24 (52.2)	39 (52.7)	0.98 (0.47–2.05)	0.955
BMI ≥ 30 kg/m^2^, n (%)	19 (15.8)	6 (13.0)	13 (17.6)	0.70 (0.25–2.00)	0.511
High school or graduation, n (%)	65 (54.2)	24 (52.2)	41 (55.4)	0.88 (0.42–1.84)	0.730
Present smoker, n (%)	11 (9.2)	5 (10.9)	6 (8.1)	1.38 (0.40–4.82)	0.611
Past smoker, n (%)	49 (40.8)	19 (41.3)	30 (40.5)	1.03 (0.49–2.18)	0.934
Ever smoker, n (%)	60 (50.0)	24 (52.2)	36 (48.6)	1.15 (0.55–2.41)	0.707
≥20 cigarettes ever in all subjects, n (%)	24 (20.0)	10 (21.7)	14 (18.9)	1.19 (0.48–2.96)	0.707
≥20 years smoke ever in all subjects, n (%)	36 (30.0)	12 (26.1)	24 (32.4)	0.74 (0.32–1.67)	0.462
Coffee drinkers, n (%) Coffee cups/day ≥2, n (%) Coffee cups/day ≥4, n (%)	107 (89.2) 92 (76.7) 21 (17.5)	42 (91.3) 38 (82.6) 12 (26.1)	65 (87.8) 54 (73.0) 9 (12.2)	1.45 (0.42–5.02) 1.76 (0.70–4.41) 2.55 (0.98–6.65)	0.554 0.228 0.056 ^
Phototype 1 and 2, n (%) Nevi ≥50, n (%)	71 (59.2) 60 (50.0)	29 (63.0) 25 (54.3)	42 (56.8) 35 (47.3)	1.30 (0.61–2.77) 1.33 (0.63–2.78)	0.496 0.453
Sunburns ever lifelong, n (%)	109 (90.8)	41 (89.1)	68 (91.9)	0.72 (0.21–2.52)	0.611
MetM, n (%) NMetM, n (%)	53 (44.2) 67 (55.8)	29 (63.0) 17 (37.0)	24 (32.4) 50 (67.6)	3.55 (1.64–7.69) 0.28 (0.13–0.61)	0.001 0.001
Stage I, n (%)	48 (40.0)	11 (23.9)	37 (50.0)	0.31 (0.14–0.71)	0.005
Stage II, n (%)	19 (15.8)	6 (13.0)	13 (17.6)	0.70 (0.25–2.00)	0.509
Stage III, n (%)	33 (27.5)	14 (30.4)	19 (25.7)	1.27 (0.56–2.86)	0.570
Stage IV, n (%)	20 (16.7)	15 (32.6)	5 (6.8)	6.68 (2.23–20.0)	<0.001
M0	100 (83.3)	31 (67.4)	69 (93.2)	0.15 (0.05–0.45)	<0.001
M1a	3 (2.5)	3 (6.5)	0 (-)	1.07 (0.99–1.15)	0.054 ^
M1b	0 (-)	0 (-)	0 (-)	-	-
M1c	17 (14.2)	12 (26.1)	5 (6.8)	4.87 (1.59–14.9)	0.003
M1d	0 (-)	0 (-)	0 (-)	-	-
Trunk, n (%)	67 (55.8)	23 (50.0)	44 (59.5)	0.68 (0.32–1.43)	0.310
Upper limb, n (%)	8 (6.7)	2 (4.3)	6 (8.1)	0.51 (0.10–2.67)	0.709
Lower limb, n (%)	26 (21.7)	10 (21.7)	16 (21.6)	1.01 (0.41–2.46)	0.988
Hands/feet, n (%)	8 (6.7)	5 (10.9)	3 (4.1)	2.89 (0.66–12.7)	0.257
Head/neck, n (%)	11 (9.2)	6 (13.0)	5 (6.8)	2.07 (0.59–7.22)	0.331
Superficial spreading, n (%)	61 (50.8)	18 (39.1)	43 (58.1)	0.46 (0.22–0.98)	0.043
Nodular, n (%)	42 (35.0)	21 (45.7)	21 (28.4)	2.12 (0.98–4.58)	0.054 ^
Acral lentiginous, n (%)	5 (4.2)	3 (6.5)	2 (2.7)	2.51 (0.40–15.6)	0.370
Lentigo maligna, n (%)	2 (1.7)	0 (-)	2 (2.7)	0.97 (0.94–1.01)	0.523
Spitzoide, n (%)	5 (4.2)	3 (6.5)	2 (2.7)	2.51 (0.40–15.6)	0.370
Breslow thickness <0.75 mm, n (%)	29 (24.2)	6 (13.0)	23 (31.1)	0.33 (0.12–0.89)	0.025
Breslow thickness ≥0.75–<1.0 mm, n (%)	11 (9.2)	5 (10.9)	6 (8.1)	1.38 (0.40–4.82)	0.747
Breslow thickness ≥1.0–<2.0 mm, n (%)	34 (28.3)	15 (32.6)	19 (25.7)	1.40 (0.62–3.14)	0.413
Breslow thickness ≥2.0–<4.0 mm, n (%)	30 (25.0)	16 (34.8)	14 (18.9)	2.29 (0.99–5.30)	0.051 ^
Breslow thickness ≥4.0 mm, n (%)	16 (13.3)	4 (8.7)	12 (16.2)	0.49 (0.15–1.63)	0.239
Ulceration, n (%)	44 (36.7)	23 (50.0)	21 (28.4)	2.52 (1.17–5.44)	0.017
Mitosis ≥1 per mm^2^, n (%)	76 (64.4) ^b^	33 (75.0) ^c^	43 (58.1)	2.16 (0.95–4.93)	0.064 ^
Regression, n (%)	15 (12.7) ^b^	5 (11.4) ^c^	10 (13.5)	0.82 (0.26–2.58)	0.735
Brisk positive TILs ^a^, n (%)	32 (27.1) ^b^	12 (27.3) ^c^	20 (27.0)	1.01 (0.44–2.34)	0.977
Non-brisk TILs ^a^, n (%)	43 (36.4) ^b^	17 (38.6) ^c^	26 (35.1)	1.16 (0.54–2.52)	0.702
TILs ^a^ absence, n (%)	42 (35.6) ^b^	14 (31.8) ^c^	28 (37.8)	0.77 (0.35–1.69)	0.509
Microsatellitosis, n (%)	5 (4.2) ^b^	3 (6.8) ^c^	2 (2.7)	2.63 (0.42–16.4)	0.360
Epithelioid variant, n (%)	34 (28.8) ^d^	7 (15.6) ^e^	27 (36.5)	0.32 (0.13–0.82)	0.014
Fusate variant, n (%)	10 (8.4) ^d^	3 (6.7) ^e^	7 (9.5)	0.68 (0.17–2.79)	0.741
Small cell variant, n (%)	2 (1.7) ^d^	0 (-) ^e^	2 (2.7)	0.97 (0.94–1.01)	0.526
More than one melanoma, n (%)	19 (15.8)	11 (23.9)	8 (10.8)	2.59 (0.95–7.04)	0.056 ^
Additional non-melanoma skin tumor, n (%)	20 (16.7)	7 (15.2)	13 (17.6)	0.84 (0.31–2.30)	0.737
Other non-skin tumor, n (%)	24 (20.0)	10 (21.7)	14 (18.9)	1.19 (0.48–2.96)	0.707

^a^ TILs, tumor infiltrating lymphocytes. ^b^ Data were available for 118 patients. ^c^ Data were available for 44 patients. ^d^ Data were available for 119 patients. ^e^ Data were available for 45 patients. Tendencies are evidenced with superscript ^.

**Table 4:** Comparison among 120 melanoma patients of clinical characteristics between the subgroup of melanoma patients having low antioxidant defense capacity (FORD < 1.10 units, n = 87) and the subgroup of melanoma patients with high antioxidant defense capacity (FORT > 1.10 units, n = 33).

Characteristic	FORD <1.10 Units (n = 87)	FORD ≥1.10 Units (n = 33)	OR (CI)	*p* Value
Dead within 5 years from study entry, n (%)	23 (26.4)	5 (15.2)	2.01 (0.69–5.83)	0.198
Age at study entry < 50 years, n (%)	15 (17.2)	6 (18.2)	0.94 (0.33–2.67)	0.904
Age at first melanoma diagnosis < 50 years, n (%)	27 (31.0)	10 (30.3)	1.03 (0.43–2.47)	0.938
Females, n (%)	38 (43.7)	11 (33.3)	1.55 (0.67–3.59)	0.303
Males, n (%)	49 (56.3)	22 (66.7)	0.64 (0.28–1.49)	0.303
All grandparents born in FVG, n (%)	65 (74.7)	23 (69.7)	1.28 (0.53–3.11)	0.579
BMI ≥ 25, kg/m^2^, n (%)	49 (56.3)	14 (42.4)	1.75 (0.78–3.93)	0.173
BMI ≥ 30 kg/m^2^, n (%)	12 (13.8)	7 (21.2)	0.59 (0.21–1.67)	0.320
High school or graduation, n (%)	50 (57.5)	15 (45.5)	1.62 (0.72–3.63)	0.238
Present smoker, n (%)	9 (10.3)	2 (6.1)	1.79 (0.37–8.75)	0.473
Past smoker, n (%)	34 (39.1)	15 (45.5)	0.77 (0.34–1.73)	0.526
Ever smoker, n (%)	43 (49.4)	17 (51.5)	0.92 (0.41–2.05)	0.838
≥20 cigarettes ever in all subjects, n (%)	19 (21.8)	5 (15.2)	1.56 (0.53–4.60)	0.416
≥20 years smoke ever in all subjects, n (%)	26 (29.9)	10 (30.3)	0.98 (0.41–2.35)	0.964
Coffee drinkers, n (%) Coffee cups/day ≥ 2, n (%) Coffee cups/day ≥ 4, n (%)	80 (92.0) 69 (79.3) 18 (20.7)	27 (81.8) 23 (69.7) 3 (9.1)	2.54 (0.78–8.22) 1.67 (0.67–4.12) 2.61 (0.71–9.53)	0.120 0.269 0.147
Phototype 1 and 2, n (%) Nevi ≥ 50, n (%)	48 (55.2) 45 (51.7)	23 (69.7) 15 (45.5)	0.54 (0.23–1.26) 1.29 (0.57–2.87)	0.151 0.540
Sunburns ever lifelong, n (%)	79 (90.8)	30 (90.9)	0.99 (0.25–3.97)	0.986
MetM, n (%)	44 (50.6)	9 (27.3)	2.73 (1.14–6.54)	0.024
NMetM, n (%)	43 (49.4)	24 (72.7)	0.37 (0.15–0.88)	0.024
Stage I, n (%)	32 (36.8)	16 (48.5)	0.62 (0.27–1.39)	0.243
Stage II, n (%)	11 (12.6)	8 (24.2)	0.45 (0.16–1.25)	0.120
Stage III, n (%)	24 (27.6)	9 (27.3)	1.02 (0.41–2.50)	0.973
Stage IV, n (%)	20 (23.0)	0 (-)	20.3 (1.19–347)	0.037
M0, n (%)	67 (77.0)	33 (100)	0.10 (0.01–1.69)	0.109
M1a, n (%)	3 (3.4)	0 (-)	1.04 (0.99–1.08)	0.560
M1b, n (%)	0 (-)	0 (-)	-	-
M1c, n (%)	17 (19.5)	0 (-)	1.24 (1.12–1.38)	0.003
M1d, n (%)	0 (-)	0 (-)	-	-
Trunk, n (%)	46 (52.9)	21 (63.6)	0.64 (0.28–1.46)	0.289
Upper limb, n (%)	4 (4.6)	4 (12.1)	0.35 (0.08–1.49)	0.213
Lower limb, n (%)	24 (27.6)	2 (6.0)	5.90 (1.31–26.6)	0.011
Hands/feet, n (%)	6 (6.9)	2 (6.0)	1.15 (0.22–6.00)	1.000
Head/neck, n (%)	7 (8.0)	4 (12.1)	0.63 (0.17–2.33)	0.492
Superficial spreading, n (%)	43 (49.4)	18 (54.5)	0.81 (0.36–1.82)	0.616
Nodular, n (%)	33 38.0()	9 (27.3)	1.63 (0.68–3.93)	0.274
Acral lentiginous, n (%)	4 (4.6)	1 (3.0)	1.54 (0.17–14.3)	1.000
Lentigo maligna, n (%)	1 (-)	1 (3.0)	0.37 (0.02–6.13)	0.476
Spitzoide, n (%)	4 (4.6)	1 (3.0)	1.54 (0.17–14.3)	1.000
Breslow thickness < 0.75 mm. n (%)	20 (23.0)	9 (27.3)	0.80 (0.32–1.99)	0.624
Breslow thickness ≥ 0.75–<1.0 mm, n (%)	8 (9.2)	3 (9.0)	1.01 (0.25–4.07)	1.000
Breslow thickness ≥ 1.0–<2.0 mm, n (%)	24 (27.6)	10 (30.3)	0.88 (0.36–2.11)	0.768
Breslow thickness ≥ 2.0–<4.0 mm, n (%)	26 (29.9)	4 (12.1)	3.09 (0.99–9.68)	0.053 ^
Breslow thickness ≥ 4.0 mm, n (%)	9 (10.3)	7 (21.2)	0.43 (0.14–1.27)	0.138
Ulceration, n (%)	32 (36.8)	12 (36.4)	1.02 (0.44–2.34)	0.966
Mitosis ≥1 per mm^2^, n (%)	56 (65.9) ^b^	20 (60.6)	1.25 (0.55–2.88)	0.591
Regression, n (%)	8 (9.4) ^b^	7 (21.2)	0.39 (0.13–1.17)	0.121
Brisk positive TILs ^a^, n (%)	24 (28.2) ^b^	8 (24.2)	1.23 (0.49–3.10)	0.661
Non-brisk TILs ^a^, n (%)	30 (35.3) ^b^	13 (39.4)	0.84 (0.37–1.92)	0.678
TILs ^a^ absence, n (%)	30 (35.3) ^b^	12 (36.4)	0.95 (0.41–2.20)	0.913
Microsatellitosis, n (%)	4 (4.7) ^b^	1 (3.0)	1.58 (0.17–14.7)	1.000
Epithelioid variant, n (%)	22 (25.6) ^c^	12 (36.4)	0.60 (0.25–1.42)	0.244
Fusate variant, n (%)	5 (5.8) ^c^	5 (15.2)	0.35 (0.09–1.28)	0.138
Small cell variant, n (%)	0 (-) ^c^	2 (6.0)	0.94 (0.86–1.02)	0.075 ^
More than one melanoma, n (%)	13 (14.9)	6 (18.2)	0.79 (0.27–2.29)	0.664
Additional non-melanoma skin tumor, n (%) Other non-skin tumor, n (%)	14 (16.1) 16 (18.4)	6 (18.2) 8 (24.2)	0.86 (0.30–2.47) 0.70 (0.27–1.85)	0.784 0.476

^a^ TILs, tumor infiltrating lymphocytes. ^b^ Data were available for 85 patients. ^c^ Data were available for 86 patients. Tendencies are evidenced with superscript ^.

**Table 5:** Comparison among 120 melanoma patients of clinical characteristics between the subgroup of melanoma patients having the combined condition of both high hydroperoxides (FORT ≥ 310 units) and low antioxidant capacity (FORD < 1.10 units) (n = 43) and the subgroup of the remaining melanoma patients (n = 77).

Characteristic	FORT ≥ 310 Units and FORD < 1.10 Units (n = 43)	All the Others (n = 77)	OR (CI)	*p* Value
Dead within 5 years from study entry, n (%)	13 (30.2)	15 (19.5)	1.79 (0.76–4.24)	0.185
Age at study entry < 50 years, n (%)	7 (9.6)	14 (18.2)	0.87 (0.32–2.37)	0.793
Age at first melanoma diagnosis < 50 years, n (%)	14 (32.6)	23 (29.9)	1.13 (0.51–2.53)	0.760
Females, n (%)	29 (67.4)	20 (26.0)	5.90 (2.61–13.4)	<0.001
Males, n (%)	14 (32.6)	57 (74.0)	0.17 (0.07–0.38)	<0.001
All grandparents born in FVG, n (%)	32 (74.4)	56 (72.7)	1.09 (0.47–2.55)	0.841
BMI ≥ 25, kg/m^2^, n (%)	23 (53.5)	40 (51.9)	1.06 (0.50–2.25)	0.871
BMI ≥ 30 kg/m^2^, n (%)	5 (11.6)	14 (18.2)	0.59 (0.20–1.77)	0.346
High school or graduation, n (%)	23 (53.5)	42 (54.5)	0.96 (0.45–2.03)	0.911
Present smoker, n (%)	5 (11.6)	6 (7.8)	1.56 (0.45–5.44)	0.488
Past smoker, n (%)	18 (41.9)	31 (40.3)	1.07 (0.50–2.28)	0.864
Ever smoker, n (%)	23 (53.5)	37 (48.1)	1.24 (0.59–2.63)	0.568
≥20 cigarettes ever in all subjects, n (%)	10 (23.3)	14 (18.2)	1.36 (0.55–3.40)	0.506
≥20 years smoke ever in all subjects, n (%)	11 (25.6)	25 (32.5)	0.71 (0.31–1.65)	0.431
Coffee drinkers, n (%) Coffee cups/day ≥ 2, n (%) Coffee cups/day ≥ 4, n (%)	39 (90.7) 35 (81.4) 11 (25.6)	68 (88.3) 57 (74.0) 10 (13.0)	1.29 (0.37–4.47) 1.54 (0.61–3.86) 2.30 (0.89–5.98)	0.687 0.362 0.087 ^
Phototype 1 and 2, n (%) Nevi ≥ 50, n (%) Sunburns ever lifelong, n (%)	27 (62.8) 24 (55.8) 38 (88.4)	44 (57.1) 36 (46.8) 71 (92.2)	1.27 (0.59–2.72) 1.44 (0.68–3.05) 0.64 (0.18–2.24)	0.546 0.342 0.488
MetM, n (%)	28 (65.1)	25 (32.5)	3.88 (1.77–8.54)	0.001
NMetM, n (%)	15 (34.8)	52 (67.5)	0.26 (0.12–0.57)	0.001
Stage I, n (%)	10 (23.3)	38 (49.4)	0.31 (0.14–0.72)	0.005
Stage II, n (%)	5 (11.6)	14 (18.2)	0.59 (0.20–1.77)	0.346
Stage III, n (%)	13 (30.2)	20 (26.0)	1.23 (0.54–2.82)	0.616
Stage IV, n (%)	15 (34.9)	5 (6.5)	7.71 (2.56–23.2)	<0.001
M0, n (%)	28 (65.1)	72 (93.5)	0.13 (0.04–0.39)	<0.001
M1a, n (%)	3 (7.0)	0 (-)	13.4 (0.68–2.66)	0.089 ^
M1b, n (%)	0 (-)	0 (-)	-	-
M1c, n (%)	12 (27.9)	5 (6.5)	5.57 (1.81–17.2)	0.001
M1d, n (%)	0 (-)	0 (-)	-	-
Trunk, n (%)	21 (48.8)	46 (59.7)	0.64 (0.30–1.36)	0.249
Upper limb, n (%)	2 (4.7)	6 (7.8)	0.58 (0.11–2.99)	0.710
Lower limb, n (%)	10 (23.3)	16 (20.8)	1.15 (0.47–2.83)	0.752
Hands/feet, n (%)	5 (11.6)	3 (3.9)	3.25 (0.74–14.3)	0.103
Head/neck, n (%)	5 (11.6)	6 (7.8)	1.56 (0.45–5.44)	0.521
Superficial spreading, n (%)	17 (39.5)	44 (57.1)	0.50 (0.23–1.05)	0.064 ^
Nodular, n (%)	20 (46.5)	22 (28.6)	2.17 (1.00–4.72)	0.048
Acral lentiginous, n (%)	3 (7.0)	2 (2.6)	2.81 (0.45–17.5)	0.348
Lentigo maligna, n (%)	0 (-)	2 (2.6)	0.97 (0.94–1.01)	0.536
Spitzoide, n (%)	3 (7.0)	2 (2.6)	2.81 (0.45–17.5)	0.348
Breslow thickness < 0.75 mm, n (%)	5 (11.6)	24 (31.2)	0.29 (0.10–0.83)	0.016
Breslow thickness ≥ 0.75–<1.0 mm, n (%)	5 (11.6)	6 (7.8)	1.56 (0.45–5.44)	0.521
Breslow thickness ≥ 1.0–<2.0 mm, n (%)	13 (30.2)	21 (27.3)	1.16 (0.51–2.63)	0.730
Breslow thickness ≥ 2.0–<4.0 mm, n (%)	16 (37.2)	14 (18.2)	2.67 (1.14–6.22)	0.021
Breslow thickness ≥ 4.0 mm, n (%)	4 (9.3)	12 (15.6)	0.56 (0.17–1.84)	0.332
Ulceration, n (%)	21 (48.8)	23 (29.9)	2.24 (1.04–4.85)	0.039
Mitosis ≥1 per mm^2^, n (%)	31 (75.6) ^b^	45 (58.4)	2.20 (0.95–5.13)	0.064 ^
Regression, n (%)	4 (9.8) ^b^	11 (14.3)	0.65 (0.19–2.18)	0.482
Brisk positive TILs ^a^, n (%)	11 (26.8) ^b^	21 (27.3)	0.98 (0.42–2.30)	0.959
Non-brisk TILs ^a^, n (%)	15 (36.6) ^b^	28 (36.4)	1.01 (0.46–2.22)	0.981
TILs ^a^ absence, n (%)	14 (34.1) ^b^	28 (36.4)	0.91 (0.41–2.01)	0.811
Microsatellitosis, n (%)	3 (7.3) ^b^	2 (2.6)	2.96 (0.47–18.5)	0.340
Epithelioid variant, n (%)	6 (14.3) ^c^	28 (36.4)	0.29 (0.11–0.78)	0.011
Fusate variant, n (%)	3 (7.1) ^c^	7 (9.1)	0.77 (0.19–3.14)	1.000
Small cell variant, n (%)	0 (-) ^c^	2 (2.6)	0.97 (0.94–1.01)	0.539
More than one melanoma, n (%)	10 (23.6)	9 (11.7)	2.29 (0.85–6.17)	0.096 ^
Additional non-melanoma skin tumor, n (%) Other non-skin tumor, n (%)	7 (16.3) 9 (20.9)	13 (16.9) 15 (19.5)	0.96 (0.35–2.62) 1.09 (0.43–2.76)	0.932 0.849

^a^ TILs, tumor infiltrating lymphocytes. ^b^ Data were available for 41 patients. ^c^ Data were available for 42 patients. Tendencies are evidenced with superscript ^.

**Table 6:** Comparison among 120 melanoma patients of clinical characteristics between the subgroup of melanoma patients having a Stage IV melanoma (n = 20) and the subgroup of the remaining melanoma patients (Stages I, II and III) (n = 100).

Characteristic	Stage IV Melanoma (n = 20)	All the Others (n = 100)	OR (CI)	*p* Value
High OS, FORT ≥ 310 units, n (%) Low TAC, FORD < 1.10 units, n (%) High OS and low TAC, FORT ≥ 310 and FORD < 1.10 units, n (%)	15 (75.0) 20 (100) 15 (75.0)	31 (31.0) 67 (67.0) 28 (28.0)	6.68 (2.23–20.0) 20.3 (1.19–347) 7.71 (2.56–23.2)	<0.001 0.037 <0.001
Dead within 5 years from study entry, n (%)	16 (80.0)	12 (12.0)	29.3 (8.40–102)	<0.001
Age at study entry < 50 years, n (%)	4 (20.0)	17 (17.0)	1.22 (0.363–4.11)	0.747
Age at first melanoma diagnosis < 50 years, n (%)	5 (25.0)	32 (32.0)	0.71 (0.24–2.12)	0.537
Females, n (%)	9 (45.0)	40 (40.0)	1.23 (0.47–3.23)	0.678
Males, n (%)	11 (55.0)	60 (60.0)	0.81 (0.31–2.14)	0.678
All grandparents born in FVG, n (%)	14 (70.0)	74 (74.0)	0.82 (0.29–2.36)	0.712
BMI ≥ 25, kg/m^2^, n (%)	10 (50.0)	53 (53.0)	0.89 (0.34–2.32)	0.806
BMI ≥ 30 kg/m^2^, n (%)	2 (10.0)	17 (17.0)	0.54 (0.11–2.56)	0.440
High school or graduation, n (%)	6 (30.0)	59 (59.0)	0.30 (0.11–0.84)	0.022
Present smoker, n (%)	3 (15.0)	8 (8.0)	2.03 (0.49–8.43)	0.330
Past smoker, n (%)	8 (40.0)	41 (41.0)	0.96 (0.36–2.55)	0.934
Ever smoker, n (%)	11 (55.0)	49 (49.0)	1.27 (0.48–3.34)	0.625
≥20 cigarettes ever in all subjects, n (%)	6 (30.0)	18 (18.0)	1.95 (0.66–5.77)	0.226
≥20 years smoke ever in all subjects, n (%)	8 (40.0)	28 (28.0)	1.71 (0.63–4.64)	0.289
Coffee drinkers, n (%) Coffee cups/day ≥ 2, n (%) Coffee cups/day ≥ 4, n (%)	18 (90.0) 14 (70.0) 5 (25.0)	89 (89.0) 78 (78.0) 16 (16.0)	1.11 (0.23–5.45) 0.66 (0.23–1.91) 1.75 (0.56–5.50)	0.895 0.442 0.338
Phototype 1 and 2, n (%) Nevi ≥ 50, n (%)	15 (75.0) 10 (50.0)	56 (56.0) 50 (50.0)	2.36 (0.80–6.99) 1.00 (0.38–2.61)	0.122 1.000
Sunburns ever lifelong, n (%)	18 (90.0)	91 (91.0)	0.89 (0.18–4.47)	0.887
M0, n (%)	0 (-)	100 (100)	0.00 (0.00–0.01)	<0.001
M1a, n (%)	3 (15.0)	0 (-)	40.2 (1.99–813)	0.016
M1b, n (%)	0 (-)	0 (-)	-	-
M1c, n (%)	17 (85.0)	0 (-)	1005 (49.7–20,316)	<0.001
M1d, n (%)	0 (-)	0 (-)	-	-
Trunk, n (%)	8 (40.0)	59 (59.0)	0.46 (0.17–1.23)	0.124
Upper limb, n (%)	1 (5.0)	7 (7.0)	0.70 (0.08–6.02)	0.745
Lower limb, n (%)	5 (25.0)	21 (21.0)	1.25 (0.41–3.85)	0.692
Hands/feet, n (%)	2 (10.0)	6 (6.0)	1.74 (0.33–9.32)	0.517
Head/neck, n (%)	4 (20.0)	7 (7.0)	3.32 (0.87–12.7)	0.079 ^
Superficial spreading, n (%)	10 (50.0)	51 (51.0)	0.96 (0.37–2.51)	0.935
Nodular, n (%)	8 (40.0)	34 (34.0)	1.29 (0.48–3.47)	0.608
Acral lentiginous, n (%)	2 (10.0)	3 (3.0)	3.59 (0.56–23.0)	0.177
Lentigo maligna, n (%)	0 (-)	2 (2.0)	0.96 (0.04–20.8)	0.980
Spitzoide, n (%)	0 (-)	5 (5.0)	0.42 (0.02–7.96)	0.566
Breslow thickness < 0.75 mm, n (%)	2 (10.0)	27 (27.0)	0.30 (0.07–1.38)	0.122
Breslow thickness ≥ 0.75–<1.0 mm, n (%)	2 (10.0)	9 (9.0)	1.12 (0.22–5.64)	0.887
Breslow thickness ≥ 1.0–<2.0 mm, n (%)	3 (15.0)	31 (31.0)	0.39 (0.11–1.44)	0.158
Breslow thickness ≥ 2.0–<4.0 mm, n (%)	9 (45.0)	21 (21.0)	3.08 (1.13–8.40)	0.028
Breslow thickness ≥ 4.0 mm, n (%)	4 (20.0)	12 (12.0)	1.83 (0.52–6.40)	0.342
Ulceration, n (%)	13 (65.0)	21 (21.0)	6.99 (2.48–19.7)	<0.001
Mitosis ≥1 per mm^2^, n (%)	14 (77.8) ^b^	62 (62.0)	1.43 (0.51–4.04)	0.499
Regression, n (%)	1 (5.6) ^b^	14 (14.0)	0.32 (0.04–2.61)	0.289
Brisk positive TILs ^a^, n (%)	6 (33.3) ^b^	26 (26.0)	1.22 (0.42–3.51)	0.712
Non-brisk TILs ^a^, n (%)	3 (16.7) ^b^	39 (39.0)	0.28 (0.08–1.00)	0.051 ^
TILs ^a^ absence, n (%)	8 (44.4) ^b^	34 (34.0)	1.29 (0.48–3.47)	0.608
Microsatellitosis, n (%)	3 (16.7) ^b^	2 (2.0)	8.65 (1.34–55.6)	0.023
Epithelioid variant, n (%)	1 (5.3) ^c^	33 (33.0)	0.11 (0.01–0.83)	0.033
Fusate variant, n (%)	1 (5.3) ^c^	9 (9.0)	0.53 (0.06–4.45)	0.561
Small cell variant, n (%)	0 (-) ^c^	2 (2.0)	0.96 (0.04–20.8)	0.980
More than one melanoma, n (%)	1 (5.0)	18 (18.0)	0.24 (0.03–1.91)	0.177
Additional non-melanoma skin tumor, n (%) Other non-skin tumor, n (%)	4 (20.0) 4 (20.0)	16 (16.0) 20 (20.0)	1.31 (0.39–4.44) 1.00 (0.30–3.32)	0.662 1.000

^a^ TILs, tumor infiltrating lymphocytes. ^b^ Data were available for 18 patients. ^c^ Data were available for 19 patients. Tendencies are evidenced with superscript ^.

## Data Availability

All data from this study are available upon reasonable request.
